# Critical Review Regarding the Application of Plant Extracts as Eco-Friendly Corrosion Inhibitors—A Sustainable Interdisciplinary Approach

**DOI:** 10.3390/molecules30183722

**Published:** 2025-09-12

**Authors:** Catalin Alexandru Barbu, Irina Fierascu, Augustin Semenescu, Cosmin M. Cotrut

**Affiliations:** 1 Faculty of Materials Science and Engineering, National University of Science and Technology Politehnica Bucharest, 313 Independentei Street, 060042 Bucharest, Romania; catalin.barbu@bnro.ro (C.A.B.); cosmin.cotrut@upb.ro (C.M.C.); 2 National Bank of Romania, Lipscani Street, 030167 Bucharest, Romania; 3 National Institute for Research & Development in Chemistry and Petrochemistry—ICECHIM Bucharest, 202 Spl. Independentei, 060021 Bucharest, Romania; 4 Faculty of Horticulture, University of Agronomic Science and Veterinary Medicine, 59 Marasti Blvd., 011464 Bucharest, Romania; 5 Academy of Romanian Scientists, Ilfov Street 3, 050044 Bucharest, Romania

**Keywords:** plant-based materials, corrosion inhibitors, active compounds, extraction methods, anti-corrosive applications

## Abstract

Corrosion remains a persistent and costly issue across multiple industrial domains, including infrastructure, transportation, and marine operations. The deterioration of metals and alloys under corrosive conditions results in significant financial losses and poses considerable environmental and safety risks. Although traditional corrosion inhibitors demonstrate high efficacy, they often contain toxic, synthetic compounds that endanger both human health and ecological systems. The increasing global focus on environmental responsibility and green chemistry has intensified the demand for alternative, non-toxic corrosion mitigation strategies. This review examines the use of plant extracts obtained through various processing methods as “environmentally friendly”, responsible corrosion inhibitors. This analysis underscores the capacity of plant-based compounds to counteract material degradation across diverse applications, from technologically advanced industrial systems to the preservation of cultural heritage. Through an interdisciplinary perspective, this review evaluates the viability of botanical approaches as sustainable substitutes for conventional inhibitors, contributing to a broader understanding of their potential across distinct fields.

## 1. Introduction

Corrosion remains a persistent and costly challenge in industrial sectors, particularly in infrastructure, transportation, and marine environments. The degradation of metals and alloys due to corrosive media not only leads to substantial economic losses, but also raises serious environmental and safety concerns [1]. Traditional corrosion inhibitors, while effective, often rely on toxic synthetic chemicals such as chromates, phosphates, and amines, which pose significant risks to human health and ecological systems. In light of the growing global emphasis on environmental sustainability and focused on green chemistry approaches, there is an urgent need to develop alternative, eco-friendly corrosion inhibition strategies.

In recent years, plant extracts have emerged as promising green corrosion inhibitors [2,3,4]. The growing pace of industrial and agricultural development has intensified the production of solid waste, posing significant threats to environmental health. At the same time, the depletion of non-renewable resources has created a pressing need for the development of sustainable, environmentally friendly, and economically viable materials for diverse applications.

Plant extracts, rich in diverse phytochemicals such as alkaloids, tannins, flavonoids, polyphenols, etc., can adsorb onto metal surfaces to form protective films that hinder electrochemical corrosion processes. The efficacy of an inhibitor is governed by its adsorption behavior, which can influence the kinetics of anodic and cathodic reactions either by sterically hindering active sites or by modifying the activation energy of the electrochemical processes. Organic inhibitors, in particular, interact with metal surfaces through donor–acceptor mechanisms—either donating electrons to vacant d-orbitals of the metal or accepting electrons into antibonding orbitals—leading to the formation of stable coordinate covalent bonds that enhance surface passivation.

These natural compounds offer several advantages: they are renewable, biodegradable, non-toxic, and often readily available as agricultural by-products [5]. Often, the plant phytochemicals can be used for the development of other types of materials, phytosynthesized metallic nanoparticles or carbon quantum dots, which can also be used as in the development of corrosive protection layers, being non-toxic and cheap and having tunable electronic properties [6,7,8]. Adopting green inhibitors helps minimize environmental harm while aligning with green chemistry principles, encouraging the use of safer and more sustainable chemical practices.

Moreover, other branches of science may become more interested in this topic when we discuss its applications in the field of cultural heritage protection [9]. The effectiveness of corrosion inhibitors should be tested and studied to select the suitable ones, especially given that for this type of application, there are some requirements which must be respected: the treatment should not noticeably alter the artifact’s surface appearance and must be as reversible as possible, allowing for future removal without causing damage. It is also crucial that the treatment preserves the original material, including any historical elements such us patinas or corrosion layers, unless they endanger the artifact’s preservation or legibility; long-term durability is essential because heritage items are designed for long-term conservation [10].

**Figure 1:**
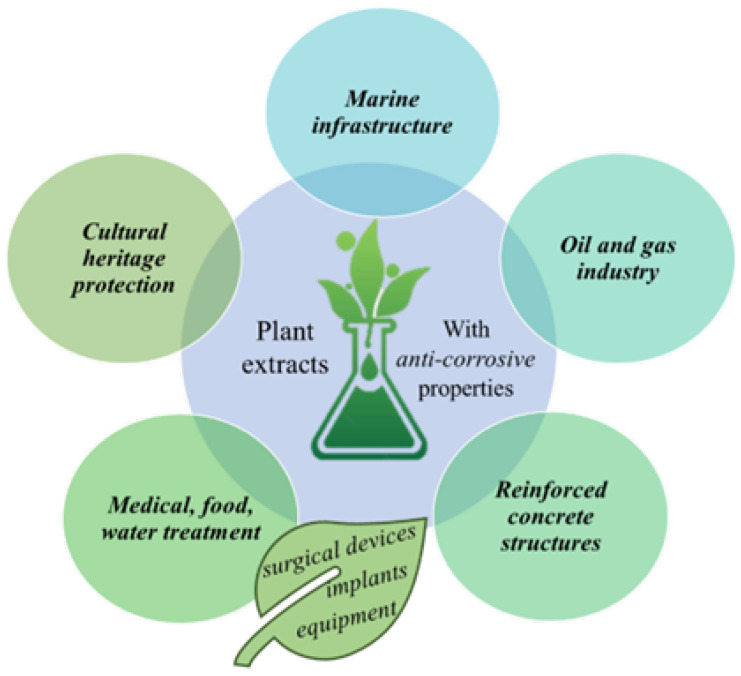
Overview of the different types of applications presented in this work.

This critical review explores the application of plant extracts, obtained through different routes, as environmentally friendly corrosion inhibitors, proving once again that the fragile world of plants can be a powerful tool against the destruction caused by corrosion processes, whether we are talking about industrial fields that offer technological progress or the protection of heritage, as an invaluable legacy for future generations. The purpose of this paper is to provide a comprehensive overview of their synthesis routes, effectiveness in various corrosive environments, and potential for industrial application, for researchers who have a basic understanding of the corrosion inhibition field but are unfamiliar with aspects related to “active molecules” responsible for the beneficial effect. Additionally, special attention is given to their use in protecting cultural heritage, our integrative approach bridging between such different domains. By integrating interdisciplinary research, this review aims to highlight the potential of plant-based approaches as sustainable alternatives to conventional corrosion inhibitors for totally different areas, thus providing a more complete picture of this topic (Figure 1) than other papers that are focused only on the study of a singular domain. The novelty of our research consists in the fact that our discussion is based only on well defined “plant extracts” and their beneficial as anti-corrosive agents, used as such, with a special emphasis on the extraction part.

## 2. Plant Extracts—A Brief Overlook, from Extraction to Application

### 2.1. Extraction Methods

As previously stated, it is important to have knowledge about extraction methods, even when dealing with extracts used for the food industry, cosmetics, agriculture, or corrosion inhibitors. In the case of plant extract-based materials, a multidisciplinary approach is needed. Some aspects of extraction methods will be discussed in relation to the proposed topic of “eco-friendly corrosion inhibitors”. Being the case of plant extract-based materials, a multidisciplinary approach is needed, and some aspects regarding extraction methods will be discussed, covering the proposed topic, “eco-friendly corrosion inhibitors”.

The selection of a solvent for extraction is primarily influenced by the solubility and volatility characteristics of the target compounds, considering the final intended applications. For instance, phenolic compounds tend to dissolve readily in polar protic solvents, while carotenoids exhibit better solubility in non-polar or polar aprotic media. The extraction of polysaccharides like pectin and hemicellulose typically involves a multi-step process using ethanol followed by alkaline treatment. In recent decades, environmentally friendly, alternative solvents have been developed to replace conventional organic solvents. These include ionic liquids, short-chain alcohols, terpenes, surfactant-based solutions, and natural deep eutectic solvents (NaDES) [11]. The selection of a suitable solvent is crucial to improve the extraction yields and, moreover, the amount of resulted waste after the extraction.

The extraction of phytochemicals with anti-corrosive properties from plant-based materials is similar to the extraction of phytochemicals with other applications and often utilizes solid–liquid techniques, with traditional methods such as Soxhlet extraction, percolation, maceration, hydro-distillation, and steam distillation also being considered into this category. However, these conventional approaches are limited by extended processing times, high volumes of solvent consumption, and often low recovery efficiency of the desired compounds. These methods present some advantages: maceration is useful for the extraction of thermolabile phytochemicals; decoction is usually applicable for the extraction of water-soluble phytochemicals and heat stable water soluble. Regarding their main disadvantages, there can be mentioned the long effective time of extraction, large amounts of used solvents, low efficiency of extraction, and large amounts of obtained wastes.

Modern extraction technologies—such as pressurized liquid extraction (PLE), microwave-assisted extraction (MAE), ultrasound-assisted extraction (UAE), and electro-technologies (high-voltage electric discharge extraction—HVED, pulsed electric field—PEF, and others)—offer substantial improvements. These approaches are known for reducing both solvent and energy use while improving extraction efficiency. These methods operate by applying other sources of energy directly to the sample, promoting compound release through other mechanisms than conventional heat transfer, being advantageous for thermally sensitive compounds. The main benefits of modern extraction techniques include higher selectivity, reduced solvent consumption, shorter processing times, as well as minimized waste generation. Nevertheless, upscaling these technologies to industrial levels often poses challenges, primarily due to the costs associated with equipment, maintenance, and auxiliary systems needed to maintain suitable operating conditions. Despite these barriers, reproducibility and process optimization are generally easier to achieve compared to lab-scale methods [12]. Tuning parameters like pressure, temperature, co-solvent presence, and extraction duration is essential to preserve the integrity of sensitive compounds, being effective for recovering a wide range of compounds (non-polar, polar) from various types of by-products, including those from cereals, winemaking, olive residues, fruit waste, etc. The risk of thermal degradation can be mitigated by adjusting solvent ratios to regulate extraction temperatures. These methods’ effectiveness often depends on the specific properties of the raw plant material. While HVED can disrupt entire cell structures and limit selectivity, PEF tends to be more targeted and is especially suitable for softer materials like peels. Rather than a single standard extraction method, many methods should be combined to enhance the extraction of active constituents [13].

**Figure 2:**
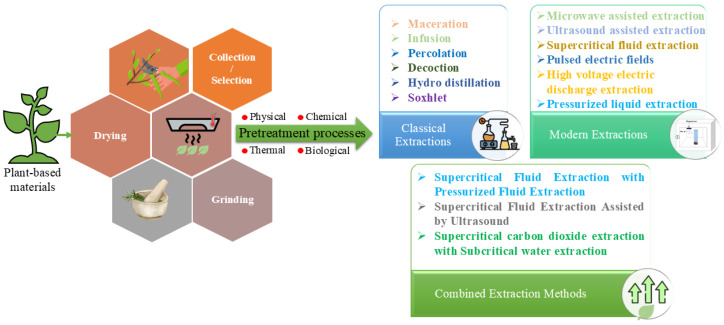
Schematic representation of the most encountered extraction methods, as presented by the studied literature data.

Environmentally sustainable extraction approaches, including the use of eco-friendly solvents, have been introduced as alternatives, aimed at minimizing waste generation, and reduce environmental pollution. These methods are generally safer and designed to have a lower ecological footprint. However, to truly assess their environmental viability, comprehensive life-cycle assessments are essential. One of the core principles of green chemistry involves lowering energy consumption through energy recovery and the adoption of innovative technologies, as well as streamlining time-intensive procedures. Nonetheless, it is important to recognize that these advanced technologies—such as microwave, ultrasound, infrared, or other electromagnetic-based methods—may actually increase overall energy demands, as they involve significant energy transfer to the extraction system. Therefore, quantifying energy use across all stages is crucial to ensure that the intended environmental benefits are realized. In Table 1 are presented advantages and drawbacks for main extraction methods, while Figure 2 present a schematic depiction of these issues, in order to obtain a wide image of the process.

To obtain new, innovative formulations, regardless of the final application (such as, but not limited to cosmetic, food, medical or industrial applications), extraction methods must be scaled up to have an increased yield and high productivity. Nowadays, with the production of a wide range of vegetal raw materials and continuous research to obtain new “eco-friendly “materials to replace hazardous chemicals, the scale-up of extraction techniques is a good opportunity, but it is not simply a matter of increasing the volume of solvents and the amount of biomass. Extensive studies have explored the transition of extraction techniques from laboratory settings to pilot and industrial scales, taking into account various factors such as equipment design, process mode (batch or continuous), reaction kinetics, cost efficiency, and energy requirements [25]. Overcoming these challenges requires thorough optimization of the equipment, which further involves designing reactors with enhanced efficiency and implementing systems that allow accurate control over energy input. Moreover, shifting different extraction methods from batch operations to continuous processes requires the simultaneous creation of dedicated continuous-flow reactors along with advanced automation and control technologies [26,27,28]. One notable example at the industrial level is the implementation of a novel ultrasound-assisted system designed for high-quality olive oil production. This setup combines ultrasonic probes with a heat exchanger to intensify the extraction process. It features three ultrasonic transducers: two rod-shaped units (30–1500 W, 30 kHz) positioned inside the processing pipe and a third (30–1400 W) located after the heat exchanger. The internal transducers mainly induce cavitation within the olive paste, which aids in breaking down cell walls and facilitates efficient oil release. The synergy between ultrasound and thermal energy contributes to a continuous, high-throughput extraction process. Additionally, precise control of flow dynamics—specifically pressure drops and fluid velocity—during ultrasound treatment has been shown to optimize the recovery of valuable components such as tocopherols, carotenoids, and phenolic compounds [28]. Some other examples of scaling-up procedures are offered in Table 2, in order to present the importance of working parameters in pilot scale/industrial extraction methods, comparable to laboratory methods, and the economic efficiency that is obtained.

In terms of feasibility, the industrial deployment of modern extraction technologies must balance technical performance with economic sustainability. Although methods such as microwave-assisted, ultrasound-assisted, or supercritical fluid extraction have demonstrated remarkable efficiency at the laboratory scale, their translation into industrial practice is highly dependent on capital investment, operational costs, and scalability of throughput. For instance, supercritical CO_2_ extraction plants designed for the food and nutraceutical industry represent an investment of EUR 1–2 million for capacities ranging from 50 to 200 L, with operating costs dominated by high-pressure equipment maintenance and CO_2_ recycling systems. Despite these costs, the technology has become commercially viable in the decaffeination of coffee and extraction of essential oils [34,35] showing that high-value applications can offset initial expenditures. For instance, in a pilot-scale UAE setup treating olive pomace (~120 kg), ultrasound-assisted extraction achieved a polyphenol yield of 3.0 g GAE/L—representing a 58% increase in extraction efficiency compared to conventional stirring-and-heating methods [36]. For corrosion inhibitor applications, the cost competitiveness will depend on the dual valorization of agro-industrial residues as raw material and the recovery of bioactive molecules with multifunctional roles (antioxidant, antimicrobial, and anti-corrosive). Since these residues are often available at low or negligible cost, the main economic driver becomes the extraction process itself. Life cycle assessment (LCA) and other green-analysis tools (such as EcoScale, GAPI, AGREE) have been increasingly applied to modern extraction techniques—notably microwave-assisted, ultrasound-assisted, supercritical fluid, and pressurized liquid extraction—to provide quantitative evaluations of environmental performance, including reductions in energy use, solvent consumption, and waste generation, compared to traditional methods such as Soxhlet or maceration [37]. Nevertheless, the feasibility of adopting such technologies for corrosion inhibitor production will require sector-specific pilot projects to evaluate not only technical performance but also cost per kilogram of extract compared to synthetic inhibitors.

Table 3 summarizes reported industrial-scale applications of modern extraction technologies, with indicative economic data. As highlighted, the main cost drivers are associated with specialized equipment, energy demand, and process maintenance, whereas the key benefits lie in reduced solvent use, shorter processing times, and higher recovery of active molecules. These examples illustrate the potential transferability to corrosion inhibitor production, particularly when valorizing low-cost agro-waste as feedstock. Nevertheless, broader industrial adoption will require further techno-economic evaluations tailored to the corrosion protection market.

### 2.2. Factors That Influence Phytochemical-Based Anti-Corrosion Treatments

Corrosion occurs when metals interact with their environment, undergoing electrochemical reactions that lead to their gradual degradation. This phenomenon poses substantial financial and operational challenges across a variety of industries. Depending on the industry type, different factors and substances can degrade various materials. In the oil and gas industry, pipelines are exposed to corrosive environments caused by common contributors that include hydrochloric acid and its derivatives, hydrogen sulfide (H_2_S), moisture, oxygen, naphthenic acids, carbon dioxide, and the interactions at petroleum–water boundaries and emulsified phases [50]. In the food processing or pharmaceutical industries, where equipment is subject to chemical and thermal stress throughout production, contamination resulting from corrosion can compromise product quality [51]. Also, in other industries such as automotive, aeronautics or civil construction problems related to corrosion are present. More corrosion-resistant alloys are available, but are not economically feasible for large-scale infrastructures. In this context, plant-derived corrosion inhibitors have emerged as effective and sustainable protection strategies, thus offering new solutions for both materials and environmental protection.

Certain plant species contain bioactive compounds that play a key role in initiating anti-corrosion activity (glycosides, alkaloids, flavonoids, phytosterol, saponins, steroids, tannins, anthraquinones, amino acids, triterpenes, phenolic compounds, etc.). These natural compounds often possess molecular and electronic characteristics comparable to those found in traditional organic corrosion inhibitors. They typically feature multiple functional groups and adsorption sites, and their molecular structures can vary from relatively simple to highly complex. One notable advantage is their richness in strongly polar organic constituents. The polar (hydrophilic) regions of these phytochemicals tend to bind to metal surfaces, facilitating adsorption, while their nonpolar (hydrophobic) segments remain in the electrolyte solution, minimizing direct interaction with the metal surface. Multiple studies have demonstrated that the key mechanism responsible for corrosion resistance involves the adsorption of molecules onto the metal surface, resulting in the formation of a chemically oriented protective layer. This layer acts as a barrier, shielding the metal from aggressive corrosive agents. This is the case of *Persea americana* Mill. leaves extract, the molecules contained being adsorbed to the metal surface naturally and uniformly, creating a protective coating [52]. The importance of extraction techniques is strongly corelated with the application, thus obtaining specific compounds that can influence anticorrosive properties. Also, the parameters of anticorrosive treatment are important.

**Figure 3:**
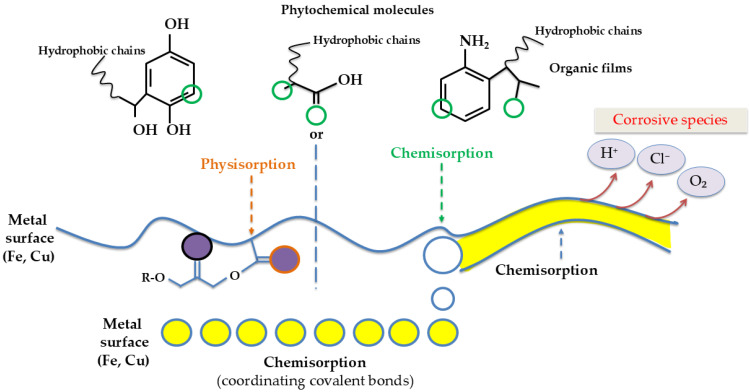
Schematic of plant extract molecules adsorbing onto metal surfaces via heteroatoms and π-systems, forming a protective organic film that blocks corrosive species (Cl^−^, H^+^).

The mechanistic action of plant extract molecules as corrosion inhibitors is based on their adsorption onto the metallic surface, where heteroatoms (O, N, S) and aromatic π-systems interact with vacant d-orbitals of the metal to form coordination bonds, while hydrophobic moieties create a barrier that restricts access of aggressive ions (Cl^−^, SO_4_^2−^, H^+^). A schematic depiction of these interactions is presented in Figure 3, summarizing the main adsorption pathways and the formation of a protective film that reduces both anodic metal dissolution and cathodic hydrogen evolution reactions.

#### 2.2.1. Temperature

Polarization study conducted at different temperatures suggested that a rise in temperature causes subsequent decrease in the inhibition efficiency of anticorrosive properties for *Magnolia grandiflora* leaves extract (applied for the protection of Q235 steel in a 1 M HCl environment) [53]. Typically, the reduction in inhibition efficiency at higher temperatures is linked to the breakdown, structural rearrangement, or decomposition of inhibitor molecules, particularly in acidic environments. In the case of application of oleuropein’s (obtained from *Olea europaea* L. leaves) anticorrosion capabilities in 1.0 M H_2_SO_4_ solutions for the treatment of copper, is quite different. The corrosion rate of copper in acid solution (whether controlled or inhibited) generally increases with rising temperature. This behavior can be attributed to surface roughening of the copper at higher temperatures, along with a shift in the adsorption–desorption equilibrium that favors the desorption of oleuropein from the copper surface. The rise of temperature maintains the inhibition ability of oleuropein constant, meaning that the oleuropein/surface system is stable at higher temperature levels [54]. In other studies, was reported that increasing the extract concentration was leading to a reduction of the corrosion rate, while higher temperatures led to an increase in corrosion. This is the case of the investigations on *Artocarpus heterophyllus* peel extract (AHPE), where the concentration was ranging from 0 to 800 ppm, on the corrosion of pure copper in HNO_3_ solution [55].

#### 2.2.2. Concentration of Plant Extract

The plant extract concentration plays a more active role than the corrosive medium in reducing the corrosion rate. However, identifying the specific bioactive molecules within the extract responsible for the inhibition proved challenging, due to their synergistic effect. An increased concentration can conduce to a better anticorrosive effect due to the activation energy at the metal/extract interface and the increased adsorption of extract molecules onto the metal surface, which limits direct contact between the metal and the corrosive environment [56]. As more extract molecules accumulate, they progressively cover the exposed surface, ultimately forming a protective layer that restricts electron transfer from the metal. This is the case of palm oil or *Pyracantha fortuneana* alcoholic extracts, used for copper as corrosion inhibitors. The efficiency was in both cases over 90%, in different corrosive media (NaCl, respectively H_2_SO_4_) at 150 ppm, respectively 600 ppm [57,58]. *Lycium barbarum* leaf extract was also efficient in H_2_SO_4_ environment with high efficiency, at 400 mg/L (92.9%, 92.8%, and 94.1% at 298 K, 303 K, and 308 K) [59]. However, an increase in extract concentration beyond a certain point does not lead to any significant improvement in the effectiveness of the plant-based extract [60].

#### 2.2.3. Immersion Time

The immersion time is an important factor, but a threshold is observed in most of the studies. According to Tian et al. [61], the initial rapid adsorption of the inhibitor molecules into the metal surface until saturation is reached usually in a certain time, and after that, the adsorption rate reduces to almost zero because of the reduced concentration of the extract [62]. In our opinion, the immersion time is in a good correlation with the extract composition, the extract being a very complex matrix depending on the species and family used, the cultivar, the geographic area, etc. It can vary from minutes or hours to days, in order to obtain a good anticorrosive efficiency [63,64].

#### 2.2.4. Composition and Molecules from Plant Extract Responsible for the Anti-Corrosive Effect

The mechanisms through which compounds from the extract can inhibit corrosion are complex and depend on the corrosive environment’s characteristics and the phytochemicals’ structure [65,66]. The main classes of phytoconstituents contains atoms such as oxygen, nitrogen, and sulfur—each with unshared electron pairs—which can form coordination bonds with vacant 3d orbitals on the surface of a material. This interaction helps reduce direct contact between the material surfaces through physical adsorption. Polar functional groups, including hydroxyl, carboxyl, and carbonyl, act as adsorption centers in this mechanism. This process also enhances the solubility of key corrosion-inhibiting agents. During the formation of a protective film on metal surfaces, heteroatoms and π-electrons contribute to adsorption, thereby preventing corrosion. Additionally, the active constituents of plant extracts often contain unsaturated bonds and aromatic rings, which may form a compact protective layer. Hydrophobic groups found in natural substances can also contribute to maintain the integrity and stability of this layer [67]. The efficiency of the inhibition is directly correlated with the strength of the bonds and layer stability [68]. In the case that the active compounds are used in different composites, resins or polymeric material, their action is correlated with the delivery rate and release rate through the synthesized protective film [69,70].

Although many studies have reported the inhibition efficiencies of crude plant extracts, more detailed investigations into structure–activity relationships (SARs) are emerging. These studies demonstrate that molecular features such as the number and position of hydroxyl groups, degree of conjugation, and presence of heteroatoms (N, O, S) are critical for anti-corrosive activity [71]. For instance, polyphenols like quercetin and gallic acid exhibit strong adsorption on mild steel surfaces due to their multiple –OH groups and aromatic π-electrons, enabling the formation of stable chemisorbed layers [72]. Alkaloids such as piperine and skimmianine provide inhibition through nitrogen lone pairs, which coordinate with metal d-orbitals, while simultaneously offering hydrophobic segments that enhance barrier properties [73]. Comparative quantum chemical studies have demonstrated that molecules exhibiting high electron-donating capacity—indicated by elevated HOMO energy levels—and those with planar, π-conjugated structures tend to interact more strongly with metal surfaces, yielding greater inhibition efficiencies. For example, phthalocyanines (macrocyclic, highly planar π-systems) exhibit efficient corrosion inhibition that is attributed to their extensive conjugation and ability to donate electrons effectively Furthermore, in alkaloid-based inhibitors, Fukui function analysis highlights the most reactive atoms that participate in surface bonding, providing a molecular-level map of donor–acceptor interactions with the metal [74,75]. These findings highlight that the anti-corrosive performance is not random but closely linked to molecular structure, and future work should systematically isolate and test individual phytochemicals to build predictive SAR models for corrosion inhibition.

While the reported studies clearly demonstrate the potential of phytochemicals as corrosion inhibitors, a critical examination reveals notable contradictions and limitations. For instance, the inhibition efficiency of extracts often decreases with rising temperature (e.g., *Magnolia grandiflora* in HCl), whereas in other cases stability at elevated temperatures is maintained (e.g., oleuropein in H_2_SO_4_), underscoring the lack of a unifying mechanism across plant species and corrosive environments [53,54]. Similarly, although higher concentrations of extracts generally improve efficiency (as seen with palm oil or *Pyracantha fortuneana* extracts), saturation effects are frequently observed, with further concentration increases providing no significant benefit [57,58]. Divergent observations are also evident regarding immersion time: while some reports indicate rapid stabilization of protective films, others suggest prolonged exposure is necessary, reflecting the complexity of extract composition and its dependence on plant variety, growth conditions, and solvent system. Even within the same family of phytochemicals, adsorption modes and film stability vary, complicating efforts to establish consistent structure–activity relationships. These contradictions highlight the current limitations in comparing literature data and stress the urgent need for standardized experimental protocols, reproducibility studies, and mechanistic investigations capable of resolving these inconsistencies.

The ability of plant extracts to prevent different materials from corrosion in different media is presented in Table 4.

The literature data also presents the synergistic effect between phytochemicals and corrosive medium [84]. For example, combining phytochemicals with small quantities of halide ions has been shown to significantly enhance the corrosion-inhibiting properties of the phytochemical compounds. In the case of effect of potassium iodide in controlling the corrosion of steel in acid medium by *Mentha pulegium* extract, the efficiency was increased from 84% to 90% [85]; meanwhile for Nettle leaves, an addition of zinc salt increased the efficiency up to 98% [86]. This improvement arises from cooperative interactions between the halides and the organic molecules, leading to the formation of a protective film on the metal surface. This film acts as a barrier against corrosive species and stabilizes the adsorption of the phytochemicals, thereby improving resistance to degradation [87].

#### 2.2.5. pH of the Environment

This parameter is an important one in the anti-corrosive treatment with active compounds obtained from vegetal material. In acid medium, metal surfaces will be positively charged due to the presence of excess hydrogen ions, while, in alkaline conditions, the metal surface will become negatively charged due to the accumulation of hydroxide ions, so active compounds, which usually present polar functional groups (e.g., –OH,–COOH), will be conducted or not towards metallic surface, depending on its charge [88]. Magni et al. demonstrated that *Punica granatum* L. peel extract have a higher efficiency at acidic levels (3–5) and decrease with the increase of pH [89].

### 2.3. Plants Extract with Anti-Corrosive Properties in Industrial Applications

The durability and long-term sustainability of industrial infrastructure is a critical challenge in civil and materials engineering, in material science, or in environmental sciences, in the face of persistent exposure to aggressive stressors, following the tendency to find new solutions, in order to keep a “clean environment” for us and for future generations. Annually, all over the world, economical losses are caused by corrosion, with an impact of billions/year, from equipment failure, loss of functionality, maintenance costs or safety hazards, highlighting the need for effective corrosion control measure [90].

In order to develop new strategies, plant extracts with anti-corrosive properties can be used as a treatment for different installation and equipment, in order to remove corrosion products or as preventive treatment, or can be use as anti-corrosive additive for different substances which circulate through pipes or installations and which can degrade them. *Psidium guajava* L. leaf extract was used by Fernandes et al. for biodiesel additivities, harnessing its antioxidant properties, thus preventing formation of corrosion products on the surface of the copper (2520 h contact at room temperature, 1440 h at 60 °C) [91].

#### 2.3.1. Marine Infrastructure

Marine infrastructure, such as offshore platforms, pipelines, and naval vessels, operates under constant exposure to harsh environmental factors—including high salinity, moisture, ultraviolet (UV) radiation, and biological contamination—that collectively accelerate corrosion and structural deterioration thus developing huge damages to this industry [92]. These aggressive conditions compromise the longevity and integrity of critical structures, resulting in frequent maintenance needs, increased operational costs, and significant financial losses. Beyond the economic burden, material degradation poses serious safety concerns and operational inefficiencies, highlighting the urgent demand for advanced, durable, and sustainable corrosion protection strategies. When talking about the use of anti-corrosive products, it is crucial to strike a careful balance among performance, environmental compatibility, and cost. In this context, the use of plant extracts can be a good opportunity for developing materials resistant in saline environment with a proper compatibility with it. *Piper nigrum* L. extract, polysaccharide extracted from the brown algae *Fucus vesiculosus*, and *Moringa oleifera* Lam. extracts are good corrosion inhibitors in saline environment [93,94,95]. The key mechanisms by which these compounds inhibit corrosion involve their polarity, antioxidant properties, and ability to form protective films [96]. These features collectively reduce the interaction between the metal surface and aggressive species by promoting strong adsorption, creating insoluble passivating layers, enhancing resistance to variations in pH and temperature, and supporting environmentally sustainable protection strategies. Also, microbial corrosion is a crucial challenge for the durability of metallic and concrete structures in coastal areas [97]. *Lupinus arboreus* Sims (LA) gum extract was successfully used for anti-fouling, anticorrosive, and self-healing properties [98].

#### 2.3.2. Oil and Gas Industry

In the case of this important industrial sector, factors which can cause damages are based on the presence of CO_2_, H_2_S, water, elevated temperature and pressure, etc., all conducting to serious economic losses. For their treatment, active compounds from plants are suitable due to their organic composition, featuring electron-rich connections and polar atoms. Moreover, the length of hydrophobic alkyl chains, low solubility of the corrosion inhibitor in the aqueous phase, heteroatoms with an increased number of cyclic or aromatic rings can influence the anti-corrosive treatment [99]. The bioactive constituents of natural products vary from one plant species to another, but their structures are closely related to their organic counterparts. Long-chain fatty acids, fatty alcohols, triterpene alcohols, sterols, and carbohydrates such as cellulose, lignin, xylose, and furfural from *Helianthus annus* L. seed shell methanolic extract reduced the corrosion rate of carbon steel in 1 M HCl solution, acting like a protective layer [100]. Pectin from apples proved to be an ecofriendly corrosion inhibitor for X60 pipeline steel in acid medium [101]. The effectiveness of corrosion inhibition (98%) was influenced by both the concentration of pectin (1000 ppm) and the temperature (98 °C) of the solution, with inhibition efficiency improving as either parameter increased. Moreover, the cleaning of these pipes is performed with the injection of HCl 15–28% solution, increasing the demand in finding new innovative formulations with anti-corrosive properties [102].

#### 2.3.3. Reinforced Concrete Structures

Carbon steel is widely used in reinforced concrete structures, particularly in the construction industry. However, it is vulnerable to various forms of corrosion, which can significantly compromise the structural integrity of concrete over time. These corrosive processes can result in severe consequences, including increased maintenance costs, structural failures, and potential threats to human safety. In environments rich in chloride, corrosion of steel reinforcement is a particularly pressing issue, often leading to early deterioration of concrete structures [103,104]. This problem poses substantial challenges for professionals, such as engineers, architects, and researchers, as well as resulting in major financial and safety-related implications. To combat corrosion, several strategies have been developed, including the use of corrosion-resistant steel, stainless steel rebars, cathodic protection systems, polymeric or epoxy coatings, and chemical inhibitors. Commonly used treatments—particularly nitrite-based ones like calcium nitrite—pose toxicity risks and may negatively affect concrete properties, such as by delaying setting time. Due to these drawbacks, their use has been restricted or banned in several countries, including those in the European Union and the United States [105]. As a result, the development and application of environmentally friendly or “green” corrosion inhibitors have gained interest. These eco-friendly compounds usually feature molecular structures with lone pair electrons, aromatic rings, and π-electrons, which can interact with the vacant d-orbitals of iron atoms in the steel. This interaction leads to the displacement of water molecules from the steel surface and the formation of a protective adsorbed film that hinders corrosion. Some researchers considered that extraction of phytochemicals is a procedure hard to scale up, with low efficiency of extraction, extracts can affect the characteristics of the concrete [106], so they use as a treatment plant powder instead of extracted liquid (*Zingiber officinale* Roscoe powder) [107]. Instead, Valdez-Salas and coworkers demonstrated that *A. indica* leaf extract (obtained through maceration of 150 g of fresh leaves immersed in 750 mL of distilled water at room temperature for 24 h) can be an excellent corrosion inhibitor for carbon steel reinforcements, when it is added in concrete composition (Holcim Apasco Class 40 Composite Portland Cement), even after 105 days of exposure to saline medium (3.5% NaCl), based on azidarachtine anti-corrosive action [108]. This tetranortriterpenoid possess oxygen heteroatoms, which allow its adsorption in the carbon steel surface, thus forming a chemisorbed protective film. Chloride ions can form in the concrete mass with high negative charge promoting corrosion on the metal surface of carbon steel-producing Fe^2+^ ions, which can interact with the lone pair of oxygen and the vacant π electrons of azadirachtine, forming covalent coordination bonds.

### 2.4. Plants Extracts with Anti-Corrosive Properties in Other Types of Applications (Medical, Food, Water Treatment)

In the last decades, other types of materials and applications gained interest among researchers, like medical, food, or water treatment, from the corrosion-protection topic point of view. It is a large subject (which can be reviewed by itself), because here are complex factors that can produce corrosion damages like corrosive medium (acidic, saline) and microbiological loading, which contribute to the corrosion [109]. Even it is about surgical devices, implants, or equipment for industrial processing (i.e., food or water), the principles of the anti-corrosive treatments are the same: finding new, “green”, suitable, and innovative solutions with proper application. The multifunctional nature of these plant-based compounds lies in their molecular structures. For example, hydroxyl and carbonyl groups enable both metal surface adsorption and free radical neutralization; nitrogen heterocycles interact with both microbial membranes and metal surfaces, giving rise to antimicrobial and corrosion-inhibiting actions; hydrophobic terpenes enhance water resistance on metal surfaces and disrupt bacterial lipid membranes [110,111]. Having multiple roles, anti-corrosive and antimicrobial, prolongs the efficiency of different devices, alongside decreasing biofilm formation [112].

For medical applications, the deterioration in implant material surface properties varies depending on factors such as the type of implant, its usage area, and the area’s movement. Stainless steel is widely employed in the fabrication of various medical instruments owing to its excellent properties as a biomedical material. Its corrosion resistance is a critical determinant of both the longevity and functional performance of medical devices, as well as a key factor influencing the material’s biocompatibility. Nevertheless, the complex and dynamic physiological environment of the human body presents significant challenges to the effective corrosion control of medical-grade stainless steel. Another example can be Ti6Al4V alloy, which is extensively utilized in dental, endovascular, and orthopedic applications due to its superior corrosion resistance, favorable mechanical properties, and excellent biocompatibility in physiological environments [113].

In the study of Sharma and coworkers, *Celastrus paniculatus* extract was presented only for anti-corrosive properties (more than 96% efficiency in 0.5 M H_2_SO_4_ solution at a concentration of 4000 pmm) [114]; meanwhile, *Araucaria araucana* was presented in the study of Gallia and collaborators as a multipurpose plant (antioxidant effect, 97%; anti-corrosive efficiency, more than 60% of the acid corrosion of steel) [115].

Extracts from *Salvia officinalis* L. show 96.6 % corrosion inhibition efficiency at 2.5 g/L [116]; 92 % inhibition efficiency at 1 g/L was reported for *Jasminum nudiflorum* Lindl. [117]; at 4 g/L, *Terminalia bellerica* (Gaertn.) Roxb. fruit extracts were proven to have a 91.79 % anti-corrosion efficiency [118]. Commercially available anti-corrosive chemicals often include chromates, dichromats, phosphates, and arsenates, but their replacement, especially in applications that involve human safety, is necessary [119]. Using heterocyclic compounds offers a superior corrosion prevention efficacy due to containing aromatic rings, π- and non-bonding electrons, as well as polar functional groups, which serve as adsorption centers on the substrate surface area [120], being overall a synergistic effect of phytochemicals in the same extract [121].

### 2.5. Plants Extract with Anti-Corrosive Properties in Cultural Heritage Protection

The preservation of metal artifacts is increasingly challenged by corrosion processes initiated by both outdoor and indoor pollutants. The interest of researchers in finding new solutions has gained more interest, but the challenge is enormous, due to the particularities of these objects and their cultural value. Conventional restoration materials and techniques often encounter major limitations, such as inadequate physicochemical compatibility with the artefact’s original components and difficulty in maintaining precise control during application. For each type of support (copper, silver, iron, etc.), the treatment and approaches are different. Usually, treatments are based on classical chemicals, most of the time toxic for human and environment [122].

Copper-based alloys, extensively used since antiquity, are particularly vulnerable to a form of corrosion known as “bronze disease.” This condition results in the formation of atacamite and its polymorphs, which are chiefly responsible for degrading the metal into a greenish powder. In recent years, various strategies have been explored to mitigate or delay corrosion. Among them, sustainable and renewable materials have been employed to encapsulate and gradually release corrosion inhibitors. For example, chitosan-based coatings infused with imidazolium salts have been utilized to safeguard bronze surfaces [123,124]. Similarly, coatings derived from chitosan have been loaded with benzotriazole and mercaptobenzothiazole to protect indoor artworks made of copper-containing alloys [124]. Protective coatings with active functionality have been developed using amorphous polyvinyl alcohol (PVA) matrices embedded with 2-mercaptobenzothiazole housed in layered double hydroxide nanocarriers. In addition to their effectiveness in corrosion prevention, these treatments must not affect the visual integrity of the objects [125].

For silver objects, corrosion products are in a good correlation with degrading factors [126]. Silver containing copper in solid solution is prone to internal oxidation, with copper preferentially corroding to form copper-based compounds. Understanding the surface corrosion layers is crucial not only for reconstructing the archaeological environmental conditions they were exposed to, but also for informing effective conservation strategies. Tarnishing, and subsequent polishing, leads to irreversible material loss and object damage, so different cleaning approaches were used: cleaning methods such as potassium cyanide solution (which has been abandoned because the chemicals are highly toxic) [127] or nitrocellulose coatings (degradation over time limits the effectiveness of the treatment) [128].

Ferrous archaeological artefacts buried for centuries typically develop thick corrosion layers, due to prolonged deterioration, based on different degradation compounds such as hematite, maghemite, magnetite, ferrihydrite, goethite, akageneite, lepidocrocite, ferroxyhite, etc. [129]. Acrylic-based polymers classical treatments have high adhesion capabilities, relatively good hydrophobicity, and also high rigidity; they tend to decay with time. Fluoropolymer treatments can be expensive; their synthesis requires very high temperatures, and their application presents safety hazards and archaeological complications [130]. The most commonly used corrosion inhibitor obtained from plant sources is tannic acid. Nut has several limitations because it modifies the color of metallic support, and it is not effective in time [131]. Compounds from *Brassica campestris* L. and *Emblica officinalis* L. extracts have been found to be effective in the anti-corrosive treatment and transform the unstable rust into stable oxides [132,133].

In other studies, different plant extracts were used as alternative and ecological methods. A *Jatropha curcas* L. extract applied on an Egyptian archaeological bronze mirror exhibited mixed-type corrosion inhibition at 30 ppm concentration, achieving 86% inhibition efficiency; the inhibitory efficiency decreased with increasing temperature due to the desorption of inhibitor molecules from the metallic surface [134]. *Robinia pseudoacacia* L. water extract was evaluated for a 0.5 M saline medium for 4 weeks at a concentration range from 200 to 1800 ppm [135], and *Spondias mombin* L. extract protects aluminum artefacts in sulfuric acid environments [136].

The effectiveness of corrosion inhibitors is affected by the composition of the substrate, environmental conditions, and chemical stability, which together determine both the frequency of application and the duration of protection. Protective performance varies widely among different metal artefacts: *Passiflora edulis* Sims, 1818 and *Laurus nobilis* L. offer 2–3 years of protection for bronze objects, while *Camellia sinensis* (L.) Kuntze preserves the artefacts for up to five years under dry conditions [137,138,139]. In our opinion, further studies are necessary for establishing the long-time effect.

The transition from traditional toxic inhibitors to green, biodegradable alternatives represents not only an ecological responsibility but also a scientific imperative for sustainable, long-term conservation, thus preserving the integrity of historical materials.

### 2.6. Benchmarking Plant Extracts vs. Conventional Synthetic Inhibitors

To avoid the fragmented perspective that often characterizes the literature on natural inhibitors, this subsection integrates comparative data from multiple reports, juxtaposing plant-based systems with conventional synthetic inhibitors to provide a coherent picture of relative efficiency, stability, and applicability.

Direct comparisons are essential for evaluating the true potential of plant-based extracts as corrosion inhibitors. Table 5 contrasts representative examples of plant extracts with conventional synthetic inhibitors under commonly studied conditions.

Several plant systems demonstrate efficiencies equal to or exceeding those of synthetics. For instance, papaya resin extract (PRE) reached nearly 98% inhibition of mild steel in HCl, while *Metaplexis hemsleyana* leaf extract achieved over 92% efficiency for Q420 steel in similar acidic media. Oleuropein, an isolated plant-derived molecule from olive leaves, displayed almost 99% inhibition of copper in H_2_SO_4_ even at elevated temperatures, surpassing the 94% efficiency of a pyridazine-based synthetic inhibitor reported under related conditions. These examples highlight the ability of bioactive plant compounds to form protective films through adsorption, supported by both experimental data and quantum chemical calculations.

Nonetheless, critical differences remain. Plant-derived inhibitors often face limitations in long-term stability due to biodegradability, susceptibility to UV or oxidative degradation, and variability between batches of extracts. By contrast, synthetic inhibitors—such as amines, nitrites, and phosphonates—generally offer longer shelf life, standardized compositions, and predictable performance, but they raise significant environmental and regulatory concerns due to toxicity and persistence. Cost comparisons are also inconclusive: raw plant materials (especially agro-waste) may be inexpensive, but extraction and standardization add costs; synthetic inhibitors may appear more expensive per unit but benefit from established logistics and reliable performance. Thus, while the laboratory efficiency of plant extracts is highly promising, their industrial uptake requires solutions for stability and standardization challenges, in parallel with efforts to phase out toxic synthetics.

## 3. Challenges and Future Perspectives in Scaling Up and Applying Plant Extracts with Anti-Corrosive Properties

Agricultural activities produce millions of tons of residues annually—such as crop straw, fruit peels, nutshells, and other organic by-products. Although a portion of this biomass is utilized for energy production or soil improvement, a large amount is still improperly discarded, leading to environmental issues like waste build-up, soil quality decline, and greenhouse gas emissions resulting from inadequate disposal methods [147]. Globally, 140 billion metric tons of biomass are generated annually, from which 1.3 billion tons refer to agro-industrial waste generated from the non-edible parts of plants [148]. Although the potential of raw material is very varied and rich, different challenges arise in expanding the use of phytochemical-based materials to industrial-scale application, in order to obtain maximum results. In terms of costs, as an example, the anthocyanin market in the year 2018 was over USD 500 million, which was expected to increase with a compound annual growth rate (CAGR) of 4.6% from 2019 to 2026 [149].

The benchmarking presented in Section 2.6 illustrates that plant-based inhibitors can in some cases match or even surpass the efficiency of conventional synthetics, such as the nearly 99% inhibition of copper by oleuropein compared to ~94% for a pyridazine-based inhibitor in similar media. However, the table also makes clear that this promising performance is counterbalanced by unresolved limitations: while synthetics benefit from stability, shelf life, and standardization, plant extracts remain affected by variability in composition, susceptibility to degradation, and scalability issues. These contradictions underline that the transition from laboratory efficacy to industrial practice requires addressing not only chemical efficiency but also cost, durability, and reproducibility, which remain major obstacles for green inhibitors.

The efficacy of plant extracts can vary significantly depending on factors such as plant species, growth conditions, and extraction methodologies. While extensive research has been conducted on the use of plant extracts as green corrosion inhibitors, limited attention has been given to their economic viability and potential for commercial-scale implementation. However, unfortunately, problems arise from the raw materials, as some sources are region-specific or seasonally dependent. Additionally, ensuring consistent quality and standardization across different batches remains a critical hurdle, in order to making such applications scalable and cost-effective in industrial processes [150]. A key limitation in the valorization of such waste materials is the lack of standardized biomass. This variability leads to inconsistencies in the physicochemical properties, sensory attributes, and bioactive compound profiles of bio-waste, thereby affecting its potential applications [151].

Another major challenge lies in the efficiency of extraction processes. Conventional methods, like solvent-based extraction, are often resource-heavy and poorly suited for large-scale implementation. To improve scalability, industries can turn to modern extraction techniques, which enhance efficiency and lower energy use. Also, the joint use of novel techniques can be an optimistic approach. Combining different pre-treatments, extraction methods, or solvents can increase the extraction yields. There is even a wide range of studies showing that, when it is a scaled-up production, particular attention is paid to the issues of cost control, operational simplicity, and systems continuity [152]. The integration of solvent recovery systems can further cut operational costs and reduce environmental burdens. Finding a proper solvent in correlation with target compounds is also challenging. A major obstacle in employing organic extracts as corrosion inhibitors lies in their poor solubility in polar electrolytes, particularly at elevated concentrations. In practice, it is often observed that these extracts tend to precipitate out when introduced into such polar environments [153].

Stability of the compounds in light, temperature, or pressure is an important factor, demanding rigorous researches in corroboration with the suitable application. Customizing formulations for specific operating conditions and performing rigorous field evaluations are necessary steps to ensure functional reliability. Moreover, the future of corrosion inhibition is trending toward the development of sustainable, environmentally benign solutions, integrating nanotechnology to enhance performance, engineering multifunctional protective coatings, and designing smart inhibitors capable of responding adaptively to environmental stimuli, which are constantly changing.

This lack of uniformity can lead to inconsistent inhibitory performance. Additionally, issues related to storage stability—such as degradation or oxidation of active constituents over time—pose further constraints on their practical application, particularly in long-term industrial settings. These factors must be carefully considered when evaluating the feasibility and reliability of plant-based inhibitors for corrosion protection.

Another consideration is economic feasibility. In some cases, however, it can be considered an alternative to hazardous chemicals when balanced with environmental protection, especially when large amounts of plant waste are available. While raw materials may be inexpensive or even freely available, it is essential to account for other factors that can significantly impact overall production costs. These include expenses related to transportation, additional treatment or processing steps or manufacturing methods. One of the key challenges in scaling up is securing a steady supply of competitively priced feedstock. To minimize transportation costs and reduce environmental impacts, sourcing raw materials locally is highly preferable. Market demand and pricing trends are crucial factors influencing the economic viability. As such, a thorough economic assessment—covering raw material costs, processing efficiency, production expenses, market demand, and price fluctuations—is essential to determine their overall feasibility.

Agro-food industries, as one of the world’s largest industrial sectors, can be a sector that provides raw materials for other industries, and interdisciplinary research can be conducted on value-added products [154]. Special interest must be conducted towards wastes with active compounds with complex structures, which offer enhanced anti-corrosive activity, and towards new methods of standardizing the newly obtained formulations.

It is important to recognize that not all bioactive compounds present in plant extracts possess corrosion-inhibiting properties. Consequently, determining the specific compound responsible for the inhibition effect of a given corrosion inhibitor remains a challenge. One possible approach is to isolate individual bioactive compounds and evaluate their inhibitory performance independently, as long as the associated costs are manageable. Further investigation is also needed to assess whether these compounds act effectively on their own or require synergistic interactions with others to inhibit corrosion. Also, the methodology used to report inhibitors’ performance could also be enhanced, requiring reproducibility tests that can help identify experimental inconsistencies or errors. Moreover, statistical analysis of data from various studies can facilitate more meaningful comparisons and provide deeper insights.

As the demand for sustainable and eco-friendly solutions grows, further exploration is essential. However, the transition from research to practical application needs the support of relevant stakeholders, and substantial work is needed to demonstrate the efficacy under real-world conditions before they can be widely adopted for commercial corrosion protection, considering that one of the primary limitations of natural plant extracts is their limited stability and short shelf life. Moreover, prolonged storage can render these extracts vulnerable to microbial and fungal contamination, which may compromise their effectiveness as corrosion inhibitors [155].

Another important limitation arises from the inconsistent and sometimes contradictory results reported in the literature. For example, while several extracts display remarkable inhibition efficiency under controlled laboratory conditions, their performance often decreases sharply with changes in temperature, pH, or immersion time, whereas in other cases such variables have little or no impact. Similarly, the role of extract concentration remains ambiguous: some studies report linear improvements in protection with higher doses, while others describe saturation thresholds beyond which no further benefit occurs. These discrepancies complicate the establishment of universal structure–activity relationships and hinder scalability. Taken together, these inconsistencies reflect the complexity of plant-based matrices and emphasize the necessity of harmonized testing methodologies, reproducibility studies, and cross-comparisons across different laboratories to transform promising experimental findings into reliable, industrially relevant solutions.

## 4. Conclusions

The implementation of circular economy principles in the production of corrosion inhibitors underscores the concept of sustainability by virtue of the utilization of renewable raw materials. The formulation process places emphasis on the utilization of non-toxic solvents and biodegradable ingredients, a strategy that serves to mitigate environmental concerns while maintaining the efficacy of the anticorrosive properties. These inhibitors, which are environmentally friendly, face implementation challenges, primarily due to their limited thermal and environmental resilience. Fluctuating humidity, temperature shifts, and exposure to pollutants can compromise their stability, thereby restricting their effectiveness in preserving material integrity. The issue of metal corrosion continues to be of significance from a technical, economic, and ecological standpoint. Combining research from different areas (from horticulture to engineering, from genomic to art conservation, etc.) with computational approaches and efficient design from laboratory scale to pilot level, technological progress can solve emergent issues.

Despite the extensive global biodiversity of plants, with hundreds of thousands of species identified, only a limited number have undergone systematic investigation for corrosion mitigation properties. The large-scale implementation of these products necessitates the refinement of extraction methods to ensure the consistency of bioactive constituents or future research in identifying exact mechanisms and isolate certain compounds responsible for the application results. Furthermore, the development corrosion inhibitors with good and stable properties in extreme conditions (temperature, high concentrations of corrosive media) is also needed.

Furthermore, the development of corrosion inhibitors with good and stable properties in extreme conditions (temperature, high concentrations of corrosive media) is also needed. As highlighted by the benchmarking in Section 2.6, plant-derived inhibitors can achieve efficiencies comparable to or even surpassing synthetic benchmarks, but their long-term stability and standardization remain unresolved, underscoring the dual need to exploit their environmental advantages while addressing the practical challenges that hinder industrial uptake. Addressing these challenges requires coordinated efforts among researchers, policymakers, and industry stakeholders, not only to refine extraction methods and ensure reproducibility, but also to establish regulatory frameworks and technological pathways that enable the transition from promising laboratory results to sustainable, large-scale industrial applications.

Enhancing resource efficiency through waste valorization not only supports environmental protection but also contributes to economic development. Emerging techniques—such as hybrid extraction processes that combine enzymatic, chemical, and physical methods—offer promising results due to their improved yields, practicality, and cost efficiency. Promoting stronger partnerships between academic institutions and industrial sectors, along with initiatives that support research translation, can help cultivate a sustainable and innovation-driven environment. Moreover, having knowledge regarding plant extracts with anti-corrosive efficiency, future research can obtained more complex formulations with enhanced properties.

## Figures and Tables

**Table 1:** Advantages and drawbacks for main extraction methods.

Category of Extraction Method	Extraction Method	Advantages	Drawbacks	Reference
Classic	Maceration, infusion, percolation, decoction	No special equipment needed; low investments costs; Possibility to extract active compounds at low temperatures	After extraction, large amounts of vegetal and solvent wastes are obtained	[14,15,16,17]
	Hydro-distillation (HD)	Less extraction time and solvent	Large amounts of waste; not applicable for many types of raw material; strict control of temperature in order to not damage thermolabile compounds	[17]
	Soxhlet	No special equipment needed; efficient extraction due to the interaction of the desired component with the solvent	Time consuming	[18]
Modern	Microwave-assisted extraction (MAE)	Reduced time of extraction; small amounts of used solvents	Expensive equipment comparative to classic extraction techniques Increased extraction efficiency only for polar solvents	[19]
	Ultrasound-assisted extraction (UAE)	Reduced time of extraction	Some pre-treatments are needed for increased extraction yields	[20]
	Supercritical fluid extraction (SFE)	Low extraction temperature favorable for thermolabile compounds; high extraction yields	High costs	[21]
	Pulsed electric fields (PEF)	High extraction yields; reduced time of extraction; small amounts of used solvents	Expensive equipment; suitable for some types of raw materials	[22]
	High-voltage electric discharge extraction (HVED)	High extraction yields; reduced time of extraction	Expensive equipment; suitable for some types of raw materials	[23]
	Pressurized liquid extraction (PLE)	High extraction yields; reduced time of extraction; increased extraction yields	Expensive equipment	[24]
Combined methods	The above-described methods can also be combined, presenting the advantages of main methods or improved properties			[18]

**Table 2:** Working parameters for laboratory and scaled-up extraction methods.

Vegetal Material	Parameters of Extraction Method at Laboratory Scale	Parameters of Extraction Method at Pilot or Industrial Scale	Reference
*Passiflora edulis* Sims leaves	Pressurized liquid extraction 70% ethanol, 100 °C, 5 cycles and 6 min of static time; solvent mass/feed mass ratio-4.25; extraction volume 34 mL	Pressurized liquid extraction Pilot scale; 80 °C, 2 cycles and 6 min of static time; solvent mass/feed mass ratio-4.25; extraction volume 2000 mL;	[26]
*Passiflora edulis* bagasse	Supercritical fluid extraction (SFE) solvent mass/feed mass ratio—80; T—40 °C; P-35 MPa; extraction vessel volume—5.44 × 10^−5^ m^3^; time—more than 200 min	SFE + PLE solvent mass/feed mass ratio—constant; T—75 °C volume—50 L, 200 L, and 500 L; time 90 min	[27]
	Pressurized liquid extraction (PLE) solvent mass/feed mass ratio—5 to 300; solvent ethanol 75%; T—65 and 75 °C; P—10 MPa; extraction vessel volume 2.95 × 10^−4^ m^3^; time—more than 200 min	SFE + PLE-US solvent mass/feed mass ratio—constant; volume—50 L, 200 L, and 500 L; time—60 min; T—75 °C	
*Eucalyptus globulus* bark	Supercritical fluid extraction Volume—0.5 L; P—200 bar and T—40 °C; solvent CO_2_	Supercritical fluid extraction Volume—5 L and 80 L; P—200 bar and T—40 °C; cosolvent ethanol—2.5 and 5%	[29]
*Theobroma cacao* L. leaves	Soxhlet extraction Mass—2 g dried sample; volume—200 mL; time 12 h	Microwave-assisted extraction Solvent 85% aqueous EtOH; volume—500 mL	[30]
*Lettuce sativa*	Microwave hydro-diffusion and gravity (MHG) Mass—300 g; volume—4 L; power—550 W	Microwave hydro-diffusion and gravity m—4 kg; volume—150 L; power—4000 W	[31]
*Pyrus communis* peels	Ultrasonic-assisted extraction 20 KHz frequency; volume—50 mL; power 60 W	Ultrasonic-assisted extraction Volume—150; 450; 3000 mL; power 600 W	[32]
*Gelidium sesquipedale*	Subcritical water treatment volume—500 mL; T—175 °C; time—130 min; P—50 bar	Subcritical water treatment Volume—5000 mL; T—185 °C; time—76 min; P—50 bar	[33]

**Table 3:** Practical considerations for modern extraction technologies at industrial scale.

Extraction Method	Typical Industrial Application	Capital Cost (Approx.)	Operational Cost Drivers	Reported Benefits	Limitations/Challenges	Reference
Supercritical CO_2_ extraction (SFE)	Extraction of essential oils and bioactives (e.g., from *Pistacia lentiscus*, maize, coffee)	Manufacturing cost (COM) for Pistacia lentiscus extract: USD 814–1000 per kg for pilot scale (yield ~0.30%) COM for maize stover wax: EUR 88.89/kg, potentially reduced to EUR 4.56–10.87/kg if residual biomass is used to generate electricity	High utility costs (electricity), CO_2_ recycling, equipment depreciation	Solvent-free, high-purity extracts; co-generation from residues reduces total cost; scalable from pilot to industrial	High initial manufacturing cost per kg extract when yields are low; economic viability depends heavily on biomass valorization and energy integration	[38,39,40]
Microwave-assisted extraction (MAE)	Extraction of polyphenols, pectin, and essential oils from plant biomass, including food processing by-products (e.g., olive leaves, tomato pomace)	~USD 50,000 for commercial MAE vessel	Electricity for microwave generators, solvent handling	Shorter extraction time (~1/3 of conventional hydro-distillation), lower energy usage (≈25% of conventional) for essential oil; significantly higher yields and reduced ecological footprint (≈50% lower environmental impact) for pectin	Scale limited to ~100 kg biomass; solvent dielectric properties and penetration affect efficiency	[41,42,43]
Ultrasound-assisted extraction (UAE)	Polyphenols, flavonoids, pectin, oils, and polysaccharides from agri-food by-products (e.g., olive pomace, orange peel, coffee silverskin, rapeseed oil, medicinal plants)	Pilot-scale reactors: flow cells (up to 250 L) or probe systems (3–100 L). Investment cost moderate (lower than SFE), but depends on reactor design and multiple transducers	Electricity for ultrasound transducers (100 W–3 kW), cooling systems for temperature control, mechanical agitation for uniform cavitation	Compared to conventional methods, ↑ yield (30–77% higher), ↓ extraction time (-70–76%), ↓ energy use (−88%), ↓ CO_2_ emissions (−93%). Works at low temperature (20–60 °C), protecting thermolabile compounds. Effective with green solvents (water, dilute ethanol).	Scale-up challenges: uneven cavitation energy in large volumes, noise (~65 dB) requiring shielding, parameter optimization for each matrix. Over-intensification may degrade sensitive compounds.	[44,45,46]
Pressurized liquid extraction (PLE)	Phenolic compounds, flavonoids, carotenoids, sterols, and polysaccharides from agro-food by-products (e.g., flax shives, grape pomace, tomato by-products, dairy matrices)	Laboratory-scale ASE systems widely commercialized (e.g., Dionex) at ~USD 40–70 k; pilot-scale units (10–50 L) considerably higher due to high-pressure vessels and pumps	Heating of solvents above boiling point, pressurization (10–15 MPa), solvent circulation and recovery systems	Short extraction times (10–20 min vs. hours for Soxhlet); 5–10× lower solvent consumption; yields equal or superior to Soxhlet; can use green solvents (ethanol, water) at subcritical conditions; improved selectivity	Still limited at industrial scale; batch mode common (low throughput); high capital cost relative to UAE/MAE; heat management challenges for water-based PLPW; scaling issues with uniform solvent penetration and plug flow	[47,48,49]

**Table 4:** The ability of plant extracts to prevent different materials from corrosion in different media.

Vegetal Material	Extraction Method/Parameters	Obtained Compounds	Corrosive Media	Material	Obtained Effects	References
*Lippia javanica* (Burm.f.) Spreng., leaves	Soxhlet, 200 mL of 100% acetone, 20 g plant; 3 h	Verbacoside	1 M HCl	Aluminum	Inhibition protection > 97% at 303 K; complex process involving physical and chemical adsorption processes. Contact angle > 90°. Active compounds contain oxygen atoms located at the carbonyl and hydroxyl groups capable of donating their lone pair electrons to the Al surface.	[76]
*Mangifera indica* L. (leaves)	Maceration, 500 mL ethanol; 15 g vegetal material; 3 h; room temperature.	Mangiferin, gallic acid; iriflophenone	1 M HCl	Mild steel	Increasing extract concentration and immersion time the efficiency—92% after 24 h in the presence of 1000 ppm inhibitor. Functional groups such as carboxylic, carbonyl and hydroxyl are responsible for the effect.	[77]
*Atriplex leucoclada* Boiss. (leaves)	Maceration; 100 g plant material; 100 mL of distilled water. The suspension was kept for 24 h without light exposure and then centrifuged at 12 °C for 35 min at 10,000 rpm.	Linalool, camphor, borneol, caryophyllene oxide, kaempferol 7-*O*-rhamnoside; undecylenic acid	1M HCl	Copper	Concentration 8 g/L of extract and 8 h immersion for efficiency 92% due to the oxygen-containing functional groups; the protective mechanism was based on formation of a layer.	[78]
*Carthamus tinctorius* L. (flowers)	Soxhlet; 2 g plant material; 1000 mL water	2-piperidinone, *N*-[4-bromo-*n*-butyl], 2-hexyl-1-decanol, 2-piperidone, 9-octadecenoic acid, undec-10-yonic acid, and tetradecyl ester	0.5 N HCl	Carbon steel	Inhibition efficiency of 89.56% at 2.5 g/L; inhibition is attributed to both physical and chemical adsorption. Computational results demonstrates that active compounds are responsible for the effect.	[79]
*Bidens aurea* (Aiton) Sherff (leaves)	Reflux process; 10g dry powder; 100 mL water; 1 h	5-*O*-caffeoylquinic acid, quercetin-3-*O*-rutinoside, aurone, and chalcone	0.5 M HCl	X42 carbon steel	Maximum efficiency—94%; The rate of corrosion is reduced by the presence of a variety of functional groups, including heteroatoms (O), aromatic rings, and multiple bonds; the donor–acceptor mechanism is to interact with the metal surface and build a protective layer at the carbon steel/solution interface.	[80]
*Piper chaba* Trel. & Yunck.	Reflux; methanol; 100 g plant powder	Piperine, viridiflorol, piplartine	2M H_2_SO_4_	Mild steel	Corrosion inhibition efficiency of 86.53 %; contact angle values from 88.00° to 83.20° indicate the hydrophilicity of the coating; the computational analysis demonstrated the adsorption of phytochemicals into a protective layer	[81]
*Polygonum aviculare* L. (leaves)	Maceration; methanol; 15 g plant material; 200 mL; 4 days	Chlorogenic acid, vanillic acid, caffeic acid, *trans*-cinnamic acid, *trans*-ferulic acid, o-coumaric acid, salicylic acid, hesperidin, rutin, isoquercitrin, Diosgenin, vanillin	1 M HCl	Mild steel	Inhibition efficiency—96%; good corrosion inhibition efficiency due to containing a series of functional groups such as heteroatoms, multiple bonds, and aromatic rings.	[82]
*Aegle marmelos* (L.) Corrêa leaf	Soxhlet; 25 g dry vegetal material; 350 mL ethanol; 5 h	Skimmianine, fagarine, scopoletin, flavone, imperatorin, umbelliferone	0.05 M HCl.	Zinc	Inhibition efficiency—78.95% to 99% at concentration of 2.0 g/L (extract) due to electron donating groups such as –OCH_3_ and –OH	[83]

**Table 5:** Snapshot comparison of plant-based vs. conventional synthetic inhibitors.

Use Case/Medium	Inhibitor (Type)	Typical Dose	Reported Efficiency	Stability/Long-Term Notes	Cost/Availability	Reference
Mild steel in HCl (acid cleaning)	Papaya resin extract (plant extract)	2000 ppm	98.08% (mixed-type; dominant anodic)	Forms protective film; DFT/MD indicate good adhesion and thermal stability of the adsorbed layer	Water extract; simple processing; natural variability possible	[140]
X70 steel in 1 M HCl (acid cleaning)	Ginkgo leaf extract (plant extract)	200 mg/L	>90% across 298–318 K	Film-forming; Langmuir adsorption; good performance over tested temperatures	Alcoholic extraction; plant-to-plant variability	[141]
Q420 steel in 1 M HCl (acid cleaning)	*Metaplexis hemsleyana* leaves extract (plant extract)	0.8 g/L	92.86%	Mixed-type; protective layer blocks ion diffusion; spontaneous adsorption (ΔG°ads ≈ −21 kJ/mol)	Water-based extraction; scalable concept; standardization needed	[142]
Copper in 1.0 M H_2_SO_4_ (acid cleaning)	Oleuropein (isolated plant molecule)	100 mg/L	98.92%; effective at elevated T	Outer protective layer; high-T efficiency retained; DFT supports strong adsorption	Purified active; requires isolation but offers batch consistency	[54]
Copper in 0.5 M H_2_SO_4_ (acid cleaning)	Pyridazine-based synthetic (synthetic organic)	(as reported)	94.1% at 298 K	Synthetic organics often show robust, predictable performance profiles	Established supply; typically higher shelf life and standardization	[143]
Coatings/long-term exposure	Plant-extract additives (e.g., henna)	(formulation-dependent)	High initial barrier improvement reported; case-specific	Limitations: biodegradability, photo-oxidation, storage instability; solvent choice critical	Often low raw-material cost; processing/standardization add cost	[144]
Coatings/long-term exposure	Conventional pigments (e.g., chromates, etc.) (synthetic/inorganic)	(formulation-dependent)	Excellent long-term performance historically	Toxicity/regulatory restrictions; environmental persistence	High synthesis cost; regulatory burden	[145]
General (various media)	Synthetic organics (amines, nitrites, phosphonates, etc.)	(system-dependent)	Often high and reliable in harsh environments; wide applicability	Longer shelf life and reliability vs. many naturals; dosing control needed	The literature is contradictory on relative costs: naturals sometimes higher due to processing; synthetics also described as more expensive in other contexts	[146]

## Data Availability

The original contributions presented in the study are included in the article; further inquiries can be directed to the corresponding author.

## Abbreviations

The following abbreviations are used in this manuscript:

NaDES	Natural deep eutectic solvents
PLE	Pressurized liquid extraction
MAE	Microwave-assisted extraction
HD	Hydro-distillation
UAE	Ultrasound-assisted extraction
HVED	High-voltage electric discharge extraction
PEF	Pulsed electric field
SFE	Supercritical fluid extraction
US	Ultrasound
CAGR	Compound annual growth rate
